# Recurrence timing and patterns incorporating lymph node status after neoadjuvant chemoradiotherapy plus esophagectomy for esophageal squamous cell carcinoma

**DOI:** 10.3389/fonc.2024.1310073

**Published:** 2024-03-06

**Authors:** Guihong Liu, Binbin Hu, Tao Chen, Xin Zhang, Yu Tang, Qian Chen, Huashan Shi

**Affiliations:** ^1^ Department of Biotherapy, Cancer Center, State Key Laboratory of Biotherapy, West China Hospital, Sichuan University, Chengdu, Sichuan, China; ^2^ Department of Cardiology, The First Affiliated Hospital of China Medical University, Shenyang, Liaoning, China; ^3^ Center of Biostatistics, Design, Measurement and Evaluation, Department of Clinical Research Management, West China Hospital, Sichuan University, Chengdu, Sichuan, China

**Keywords:** esophageal squamous cell carcinoma, neoadjuvant chemoradiotherapy, recurrence, lymph node status, lymph node metastasis, ypN

## Abstract

**Purpose:**

About 40% of esophageal squamous cell carcinoma (ESCC) patients experienced recurrence after neoadjuvant chemoradiotherapy (nCRT) plus esophagectomy. While limited information was available on recurrence risk stratification in ESCC after neoadjuvant treatment. Our previous study showed ypN status was a reliable tool to differentiate and predict the prognosis in the recurrent population. Here, we evaluated recurrence timing and patterns in ESCC patients, taking into consideration lymph node status after nCRT.

**Materials and methods:**

A total of 309 ESCC patients treated with nCRT plus esophagectomy between 2018 and 2021 were enrolled in this observational cohort study. Lymph node status was recorded by the pathologist according to the surgical specimens. We retrospectively investigated the timing and patterns of recurrence and the prognoses in ESCC patients, taking into consideration lymph node status after nCRT.

**Results:**

After nCRT plus surgery in ESCC patients, lymph node metastasis was associated with unfavorable clinicopathological factors and high risks of recurrence. In the recurrent subgroup, ypN+ patients experienced earlier recurrence, especially for locoregional recurrence within the first year. Moreover, ypN+ patients had poorer prognosis. However, the recurrence patterns in the ypN- and ypN+ groups were similar. Besides, there were no significant differences in surgery to recurrence, recurrence to death, or overall survival among patients with locoregional or distant recurrence for overall patients and within ypN- or ypN+ groups.

**Conclusions:**

Lymph node metastasis was correlated with unfavorable clinicopathological factors and high risks of recurrence. Despite a similar recurrence pattern in the recurrent subgroup between the ypN- and ypN+ groups, ypN+ patients exhibited earlier recurrence and a worse prognosis.

## 1 Introduction

Neoadjuvant chemoradiotherapy (nCRT) followed by esophagectomy is the standard treatment for patients with locally advanced esophageal carcinoma based on the success of the CROSS and the NEOCRTEC5010 trials (1, 2). Despite improvement in recurrence-free survival (RFS) and overall survival (OS), the risk of recurrence after nCRT remains high in esophageal carcinoma. After a long-term follow-up, the CROSS trial reported that 49% (87/178) of patients in the nCRT group experienced overall disease progression. Among them, 19.5% (17/87) had locoregional recurrence (LRR), while 80.5% (70/87) had distant metastasis (including both locoregional and distant progression) (3). In the NEOCRTEC5010 trial, the corresponding data were 33.7% (62/184), 29.0% (18/62), and 71.0% (44/62), respectively (4).

Few studies have separately analyzed the population of recurrent patients. The 8th edition of the AJCC TNM classification introduces a distinct staging system known as ypTNM stage, specifically designed for patients who have received neoadjuvant therapy. Zhou S et al. classified the group of patients with recurrence based on ypTNM stage, whereas Nagaki Y et al. categorized the recurrent population using tumor regression grade (TRG) (5, 6). In the study conducted by Nagaki Y et al., they defined TRG3 as highly effective with no evidence of viable cancer cells and observed that TRG3 patients had no locoregional recurrence (6). However, analysis from large-scale clinical studies suggested that even patients with pathological complete response (pCR) experienced locoregional recurrence (7, 8). Furthermore, there was no statistically significant difference in OS based on TRG classification among the recurrent population (6). In the study by Zhou S et al., it was observed that adjacent ypTNM stages were combined for analysis, indicating that the use of ypTNM for risk stratification in the recurrent population was questionable (5).

According to our previous research, we found that the ypN status was a reliable indicator for differentiating and predicting the prognosis of patients who experienced recurrence after undergoing nCRT followed by surgery (data not shown). “ypN status” refers to lymph node status after neoadjuvant therapy, and is further divided into “ypN-” (no residual lymph nodes) and “ypN+” (residual lymph nodes) based on the absence or presence of lymph node involvement. Researches also showed ypN status was associated with the response to nCRT, the utilization of adjuvant therapy, recurrence, and prognosis (4, 9, 10). Thus, in this study, we stratified the recurrent population based on ypN status and further analyzed recurrence timing, recurrence pattern, and prognosis.

## 2 Materials and methods

### 2.1 Patients

We conducted a retrospective analysis on ESCC patients who underwent nCRT followed by esophagectomy. These individuals were identified from a prospectively maintained database collecting the patients undergoing esophagectomy for all reasons at West China Hospital of Sichuan University from 2018 through 2021. Patients with non-R0 resection and incomplete pathological data were excluded. Clinical staging was based on the 8th edition of the AJCC TNM classification mainly according to the findings of contrast-enhanced computed tomography. Patients with a supraclavicular lymph node (cM1 lymph node) were included. This analysis received approval from the Institutional Review Board at West China Hospital (2023-1343).

### 2.2 Treatment

nCRT was used in patients with locally advanced ESCC with ECOG score of 0-1. The chemotherapy regimen consisted of either an intravenous or oral fluoropyrimidine, or a taxane, with or without a platinum compound. The oncologists determined the specific chemotherapy regimen and the number of cycles based on their clinical judgment and the patient’s condition. Patients underwent radiotherapy at a prescribed dose ranging from 40.0 to 50.4 Gy, using either intensity-modulated or three-dimensional conformal radiotherapy techniques. The radiotherapy target was delineated around the gross tumor volume and metastatic lymph nodes, with appropriate margins as per the guidelines. Anatomical structures such as the heart, lungs, bones, kidneys, and liver were avoided during radiotherapy.

Surgery was performed at least one month after completing nCRT, by which time patients had no treatment-related adverse events worse than 2 according to the Common Terminology Criteria for Adverse Events (CTCAE). All patients underwent minimally invasive McKeown esophagectomy, which includes en-bloc esophagectomy and complete thoraco-abdominal two-field lymph node dissection (LND) as the standard procedure (11). Three-field LND was only performed for patients with highly suspected cervical nodal disease.

### 2.3 Pathological analysis

A skilled pathologist examined the surgical specimens and determined the stage based on the 8th edition of the AJCC TNM classification. R0 means no tumor cells were found within 1 mm of the surgical margins. If tumor cells are present within 1 mm of the margins, it is classified as R1. R2 indicates visible remaining tumor tissue. The primary tumor was examined for histological features like lymphovascular invasion, perineural invasion, and treatment response. The response of the tumor to nCRT was evaluated using the College of American Pathologist (CAP) Cancer Protocol for Esophageal Carcinoma. The Tumor Regression Grade (TRG) is categorized into four groups: TRG 0 (complete response, no cancer cells remaining), TRG 1 (near complete response, minimal remaining cancer cells), TRG 2 (partial response, partial shrinkage of the tumor), and TRG 3 (poor response, significant remaining tumor).

### 2.4 Follow-up and treatment after recurrence

Patients were scheduled for regular surveillance following surgery, with appointments every 3 months for the first 2 years, every 6 months for the next 3 years, and then annually. During these visits, patients underwent physical examinations, blood tests, and enhanced CT scans, typically covering the head, neck, thorax, and abdomen. Additional specialized tests were arranged when necessary. In cases of recurrence, treatment options included chemotherapy, radiotherapy, and/or immunotherapy, with some patients also participating in clinical trials. OS was defined as the period from the surgery date until death or the last follow-up, while DFS referred to the time between surgical resection and the first instance of recurrence, death, or the last follow-up. Depending on where recurrence first occurred, relapses were classified as locoregional or distant. Locoregional recurrences were defined as recurrences at the site of the primary tumor or locoregional lymph nodes, including lymph node recurrences at the celiac trunk. Distant recurrences were defined as extra-regional lymph node metastasis, systemic metastases, or spread to pleural or peritoneal regions. Lymph node recurrences in the supraclavicular region were considered to be distant.

### 2.5 Statistical analysis

Continuous variables with a normal distribution were presented as mean ± standard deviation. To evaluate differences between groups, the One-way ANOVA test was employed. For continuous variables with a skewed distribution, median with a range (minimum-maximum) was used, and the differences were analyzed using the Kruskal-Wallis or Mann-Whitney test. Categorical data were compared between groups using Chi-square tests. Clopper & Pearson exact test was used for calculating the 95% confidence intervals of the single sample rate. Survival probabilities were calculated using the Kaplan-Meier method, and differences in survival rates were assessed using the log-rank test. The strength of the relationship was determined by odds ratios, along with 95% confidence intervals (CIs). Statistical analyses were performed using IBM SPSS Statistics 25.0, the R statistical software, and GraphPad Prism 9.5.0 software. Statistical significance was defined as *P* < 0.05.

## 3 Results

### 3.1 Lymph node status and patient characteristics

**Figure 1:**
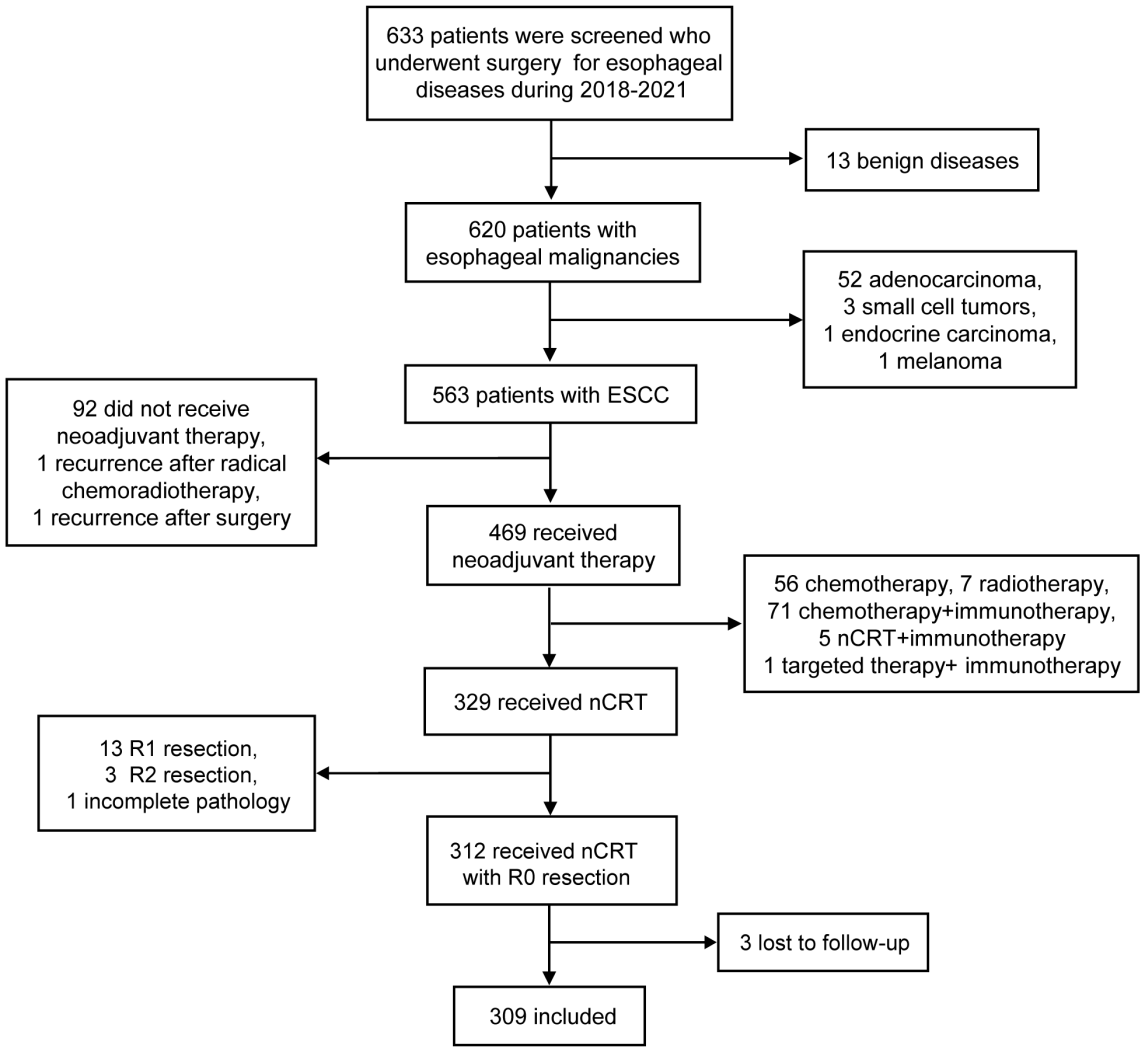
Flowchart of included patients. ESCC, esophageal squamous cell carcinoma; nCRT, neoadjuvant chemoradiotherapy.

A total of 633 patients who underwent esophagectomy for any reason at our institution between 2018 and 2021 were screened. Out of the 633 screened patients, 563 had ESCC and 329 of them received nCRT followed by esophagectomy. Among these patients, 3 patients underwent R2 resection, 13 patients underwent R1 resection and 1 patient had incomplete pathological data. Excluding 3 patients lost to follow-up, 309 patients were included in this study (**Figure 1**). The loss to follow-up rate was 0.96% (3/312 patients). They were further categorized into ypN- (212 patients, 68.6%) or ypN+ (97 patients, 31.4%). Their characteristics are shown in **Table 1**. There were no differences among groups with respect to age, ECOG and NRS score, smoking history, tumor location and length, radiotherapy dose, cT and cM stage, and number of LND. In comparison with patients with ypN+, ypN- patients had a higher proportion of females (*P* = 0.013), non-drinkers (*P* = 0.008), and an earlier clinical stage (*P <*0.001 for cN stage, *P* = 0.002 for cTNM stage). Moreover, ypN status was associated with primary tumor response to nCRT. The proportions of ypN- group with TRG0-3 were 59.0%, 15.6%, 24.5%, and 0.9% respectively, while these proportions in the ypN+ group were 18.6%, 23.7%, 49.5%, and 8.2% (*P* < 0.001). The ypN- group also had a higher likelihood of being negative for lymphovascular and perineural invasion (*P* < 0.001) (**Table 1**).

**Table 1:** Correlation between lymph node status, clinicopathological factors, and prognosis.

	ypN-	ypN+	p value
**Number**	212 (68.6%)	97 (31.4%)	
**Sex**			0.013
Female	47 (22.2%)	10 (10.3%)	
Male	165 (77.8%)	87 (89.7%)	
**Age**			0.945
<60	80 (37.7%)	37 (38.1%)	
≥60	132 (62.3%)		
**PS**			0.161
0	158 (74.5%)	69 (71.1%)	
1	53 (25.0%)	25 (25.8%)	
2	1 (0.5%)	3 (3.1%)	
**NRS**			0.968
0	136 (64.2%)	62 (63.9%)	
≥0	76 (35.8%)	35 (36.1%)	
**Smoking History**			0.214
NO	93 (43.9%)	33 (34.0%)	
YES	88 (41.5%)	50 (51.5%)	
YES but quit	31 (14.6%)	14 (14.4%)	
**Alcohol History**			0.008
NO	102 (48.1%)	31 (32.0%)	
YES	91 (42.9%)	60 (61.9%)	
YES but quit	19 (9.0%)	6 (6.2%)	
**Tumor Location**			0.79
Upper	17 (8.0%)	6 (6.2%)	
Middle	133 (62.7%)	60 (61.9%)	
Distal	62 (29.2%)	31 (32.0%)	
**Barium_Length, cm**	5.214±1.806	5.327±1.924	0.617
**Radiotherapy Dose, cGy**			0.335
≤40Gy	91 (42.9%)	36 (37.1%)	
>40Gy	121 (57.1%)	61 (62.9%)	
**cT Stage**			0.897
cT1	1 (0.5%)	0 (0.0%)	
cT2	30 (14.2%)	13 (13.4%)	
cT3	139 (65.6%)	63 (64.9%)	
cT4	42 (19.8%)	21 (21.6%)	
**cN Stage**			<0.001
cN0	41 (19.3%)	1 (1.0%)	
cN1	83 (39.2%)	42 (43.3%)	
cN2	78 (36.8%)	39 (40.2%)	
cN3	10 (4.7%)	15 (15.5%)	
**cM Stage**			0.355
cM0	193 (91.0%)	85 (87.6%)	
cM1	19 (9.0%)	12 (12.4%)	
**cTNM Stage**			0.002
II	50 (23.6%)	8 (8.2%)	
III	106 (50.0%)	50 (51.5%)	
IV	56 (26.4%)	39 (40.2%)	
**Number of lymph node dissection**			0.830
≥15	194 (91.5%)	88 (90.7%)	
<15	18 (8.5%)	9 (9.3%)	
**TRG**			<0.001
0	125 (59.0%)	1 (18.6%)	
1	33 (15.6%)	23 (23.7%)	
2	52 (24.5%)	48 (49.5%)	
3	2 (0.9%)	8 (8.2%)	
**Lymphovascular invasion**			<0.001
NO	208 (98.1%)	66 (68.0%)	
YES	0 (0.0%)	23 (23.7%)	
uncertain	4 (1.9%)	8 (8.2%)	
**Perineural invasion**			<0.001
NO	186 (87.7%)	65 (67.0%)	
YES	23 (10.8%)	28 (28.9%)	
uncertain	3 (1.4%)	4 (4.1%)	
**Recurrence**			<0.001
Absence	166 (78.3%)	40 (41.2%)	
Presence	46 (21.7%)	57 (58.8%)	
**Prognosis**			<0.001
Alive	166 (78.3%)	35 (36.1%)	
Dead with ESCC	42 (19.8%)	56 (57.7%)	
Dead with other diseases	4 (1.9%)	6 (6.2%)	

### 3.2 Survival analysis of the recurrence

All patients were observed until July 2023, with a median follow-up duration of 39.2 months (range, 0.6-70.4 months). Recurrence was detected in 33.3% (103 patients) of all patients, including 21.7% (46/212) of ypN- patients and 58.8% (57/97) of ypN+ patients (*P* < 0.001) (**Table 1**). The recurrence time and location of 5 patients were unrecorded. In total, the recurrence analysis included 98 patients. As we have described previously, the survival curves of the recurrence for OS differentiated based on the status of ypN (data not shown). The OS of the ypN- group was significantly longer compared to that of the ypN+ group (HR: 1.819, 95%CI: 1.150-2.878, *P* = 0.011). The 1-year, 2-year, and 3-year OS rates for the ypN- group were 86.4%, 47.2%, and 27.1% respectively. In the ypN+ group, the respective rates were 63.0%, 25.9%, and 16.7% (**Table 2**).

**Table 2:** Overall survival rate of patients with or without lymph node metastasis.

	No. Patients	Frequency, %	1-yr OS (95%CI)	2-yr OS (95%CI)	3-yr OS (95%CI)	Hazard Ration (95%CI)	P-value
ypN	98	100.0	73.5 (64.7,82.3)	35.4 (25.8,45.0)	21.0 (12.4,29.6)	—	—
ypN-	44	44.9	86.4 (76.2,96.6)	47.2 (32.3,62.1)	27.1 (12.8,41.4)	Reference	
ypN+	54	55.1	63.0 (50.1,75.9)	25.9 (14.1,37.7)	16.7 (6.1,27.3)	1.819 (1.150-2.878)	0.011

**Figure 2:**
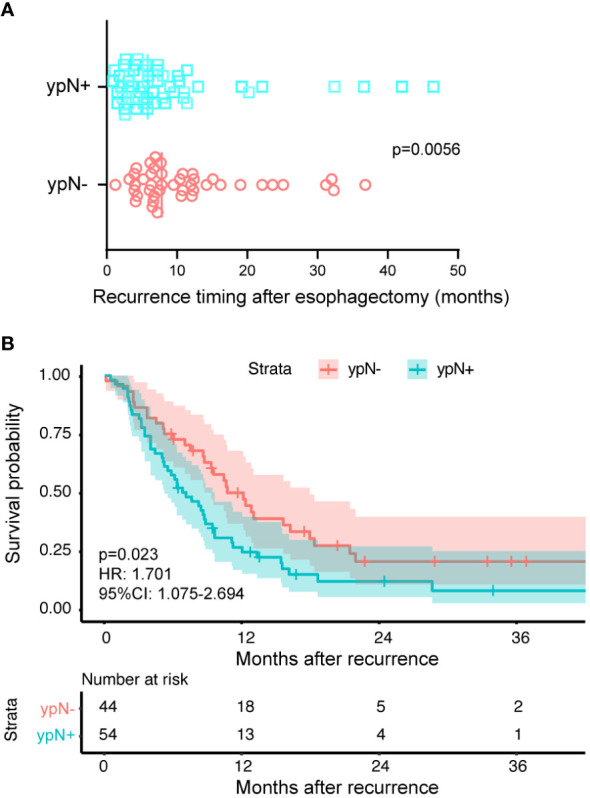
The reasons attributed to varied prognosis induced by lymph node status. The ypN- and ypN+ groups had 44 and 54 patients, respectively. **(A)** The time from surgery to recurrence classified according to the ypN status. The symbols represent individual patients who experienced recurrence, while the vertical solid lines indicate the median values. The ypN- group had a median recurrence time of 7.8 months after surgery, compared to 5.8 months in the ypN+ group (*P* = 0.0056). **(B)** Kaplan-Meier survival curves comparing the time from recurrence to death between ypN- and ypN+ patients. The log-rank test was used to compare the curves. Compared to the ypN+ group, the ypN- group had a longer OS (HR: 1.701, 95%CI: 1.075-2.694, *P* = 0.023).

The difference in OS between the two groups consisted of two components: the time from surgery to recurrence and the time from recurrence to death. The median time from surgery to recurrence was 7.8 months in the ypN- group and the value was 5.8 months in the ypN+ group (*P* = 0.0056) (**Figure 2A**). From **Figure 2B**, it can be observed that the ypN- group had a longer OS even after recurrence compared to the ypN+ group (HR: 1.701, 95%CI: 1.075-2.694, *P* = 0.023).

### 3.3 Recurrence pattern

**Figure 3:**
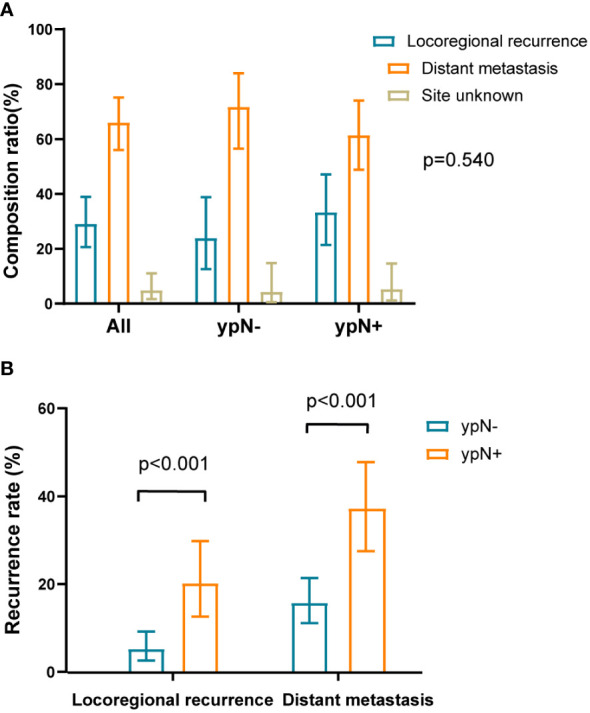
The composition ratio and recurrence rate of local recurrence and distant metastasis. **(A)** The composition ratios of local recurrence and distant metastasis in overall patients and within ypN- or ypN+ subgroups. The distribution of recurrence between the ypN- and ypN+ groups were similar (*P* = 0.540). The sum of composition ratios may not be equal to 1 due to rounding. **(B)** The recurrence rates of local recurrence and distant metastasis in recurrent population divided by the ypN status. Compared to the ypN- group, the ypN+ group had higher rates of locoregional recurrence (*P* < 0.001) and distant metastasis (*P* < 0.001).

Among the overall patients, 68 patients (66.0%) had distant recurrence, 30 (29.1%) had locoregional recurrence, and 5 (4.9%) had an unknown site of recurrence. In the ypN- group, the proportions for distant recurrence, locoregional recurrence, and unknown site of recurrence were 71.7%, 23.9%, and 4.3%, respectively. In the ypN+ group, the proportions were 61.4%, 33.3%, and 5.3%, respectively. There was no difference in the distribution of recurrence between the ypN- and ypN+ groups (*P* = 0.540) (**Figure 3A**). Excluding 5 unknown recurrent sites (2 ypN- and 3 ypN+), the rate of locoregional recurrence was 5.2% (11/210) in the ypN- group and 20.2% (19/94) in the ypN+ group. The rate of distant metastasis was 15.7% (33/210) in the ypN- group and 37.2% (35/94) in the ypN+ group. All differences were statistically significant with a P-value < 0.001 (**Figure 3B**).

**Figure 4:**
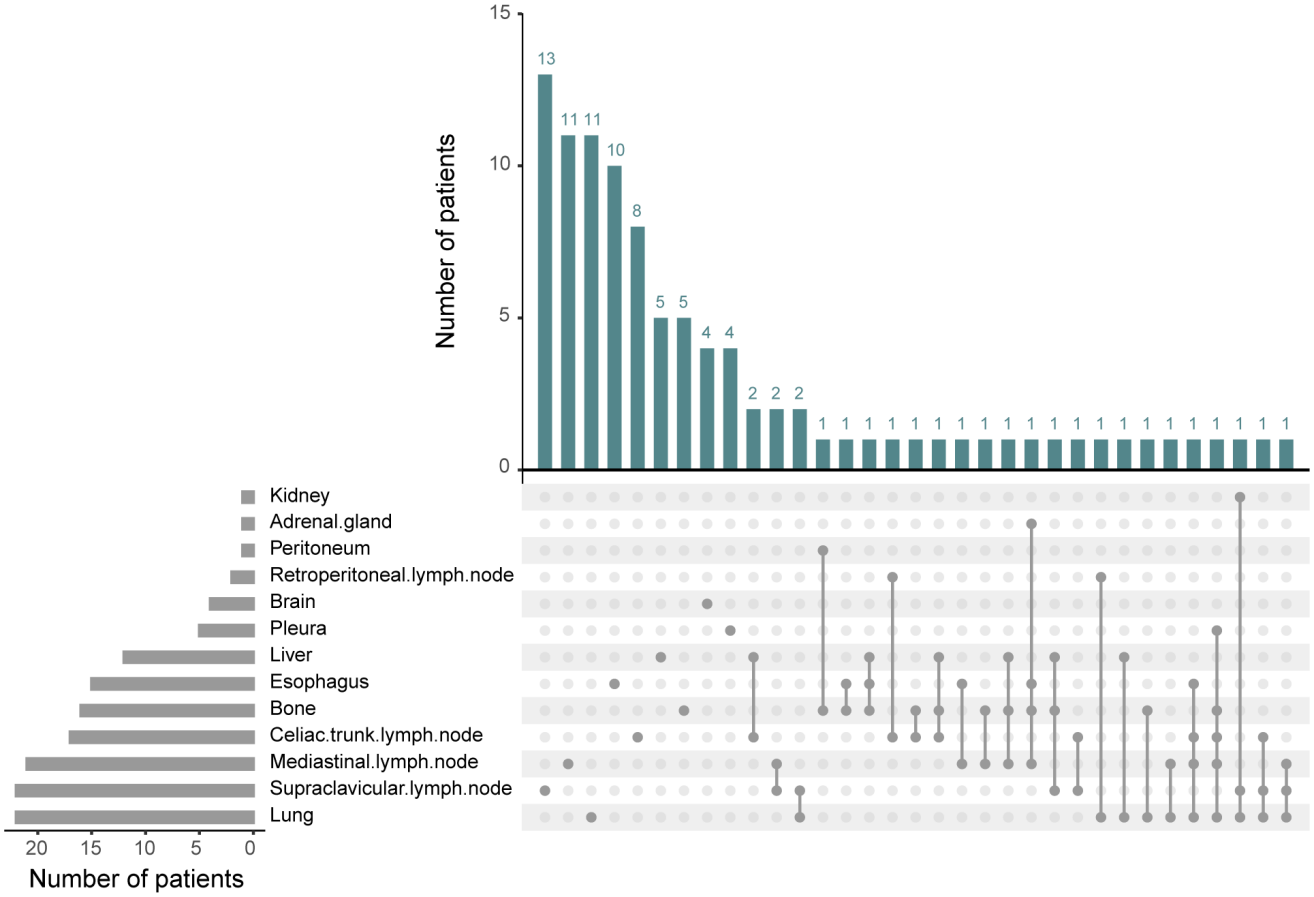
The recurrence sites after neoadjuvant chemoradiotherapy plus esophagectomy. Among the 98 recurrent patients, a total of 13 recurrence sites were observed. The predominant locations included lung, supraclavicular lymph node, mediastinal lymph node, celiac trunk lymph node, bone, esophagus, and liver.

Out of 103 patients with recurrence, the site of recurrence was known for 98 individuals. As was shown in **Figure 4**, recurrence occurred most frequently in the lung (22.4%, 22/98) and supraclavicular lymph node (22.4%, 22/98), followed by mediastinal lymph node (21.4%, 21/98), celiac trunk lymph node (17.3%, 17/98), bone (16.3%, 16/98), esophagus (15.3%, 15/98), liver (12.2%, 12/98), pleura (5.1%, 5/98) and brain (4.1%, 4/98). Recurrence in retroperitoneal lymph node, peritoneum, adrenal gland, and kidney was relatively rare. We then explored the specific recurrence site after esophagectomy for ESCC that took into consideration of ypN status after nCRT. Site recurrence rates in the ypN- and ypN+ patients were shown in **Table 3**. There was no difference in the site of recurrence between the two groups, except for brain metastasis.

**Table 3:** Distribution of recurrence sites by lymph node status.

Site of recurrence	ypN-(N=44)	ypN+(N=54)	p value
Locoregional			
Esophagus	4 (9.1%)	11 (20.4%)	0.123
Mediastinal lymph node	9 (20.5%)	12( 22.2%)	0.832
Celiac trunk lymph node	5 (11.4%)	12 (22.2%)	0.188
Mutiple sites	0 (0.0%)	1 (1.9%)	1
Distant			
Supraclavicular lymph node	11 (25.0%)	11 (20.4%)	0.632
Retroperitoneal lymph node	0 (0.0%)	2 (3.7%)	0.5
Lung	9 (20.5%)	13 (24.1%)	0.429
Liver	3 (6.8%)	9 (16.7%)	0.216
Kidney	0 (0.0%)	1 (1.9%)	1
Adrenal gland	0 (0.0%)	1 (1.9%)	1
Pleura	3 (6.8%)	2 (3.7%)	0.654
Peritoneum	0 (0.0%)	1 (1.9%)	1
Brain	4 (9.1%)	0 (0.0%)	0.038
Bone	7 (15.9%)	9 (16.7%)	1
Mutiple organs	8 (18.2%)	18 (33.3%)	0.11

### 3.4 Recurrence pattern-related recurrence timing and prognosis

**Figure 5:**
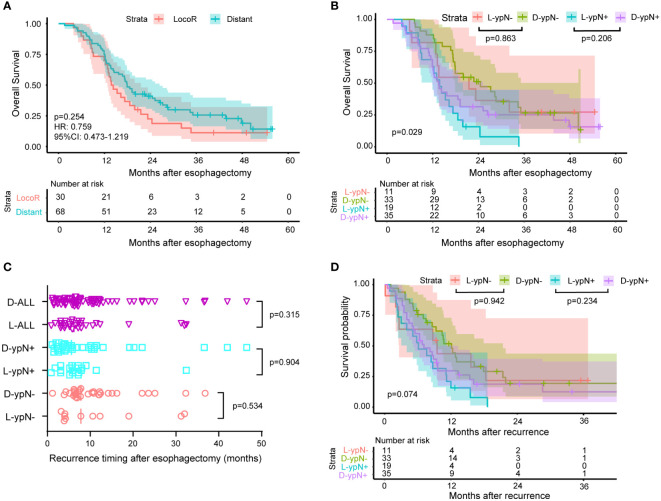
Survival and recurrence timing between patients with locoregional recurrence and patients with distant metastasis. 30 patients experienced locoregional recurrence and 68 patients had distant metastasis. Out of the 44 ypN- patients who experienced recurrence, 11 had locoregional recurrence, and 33 had distant metastasis. In the ypN+ group, these numbers were 54, 19, and 35, respectively. **(A)** Kaplan-Meier survival curves comparing OS between patients with locoregional and distant recurrence. No significant difference in OS was observed between patients with locoregional recurrence and those with distant recurrence (HR: 0.759, 95% CI: 0.473-1.219, *P* = 0.254). **(B)** Kaplan-Meier survival curves comparing OS between patients with locoregional (L) and distant (D) recurrence within ypN- or ypN+ subgroup. No significant differences in OS were detected between patients with locoregional recurrence and those with distant recurrence within the ypN- and ypN+ groups (*P* = 0.863 for ypN-, *P* = 0.206 for ypN+). **(C)** The time from surgery to recurrence classified according to the ypN status and recurrence pattern. The median time to the first recurrence was similar between the locoregional (L) and distant (D) recurrence groups for all patients (*P* = 0.315), the ypN- group (*P* = 0.534), and the ypN+ patients (*P* = 0.904). **(D)** Kaplan-Meier survival curves comparing the time from recurrence to death between patients with locoregional (L) and distant recurrence (D) within ypN- or ypN+ subgroup. The log-rank test was used to compare the Kaplan-Meier survival curves. The survival probability after recurrence was comparable between the locoregional and distant recurrence groups in both the ypN- group (*P* = 0.942) and the ypN+ group (*P* = 0.234).

The Kaplan-Meier survival curves were plotted according to locoregional and distant recurrence (**Figure 5A**). For the recurrent patients, there was no significant difference in OS between the locoregional and distant recurrence groups (HR: 0.759, 95%CI: 0.473-1.219, *P* = 0.254). Similarly, within the ypN- and ypN+ groups, there were no significant differences in OS between patients with locoregional recurrence and those with distant recurrence (*P* = 0.863 for ypN-, *P* = 0.206 for ypN+, **Figure 5B**). Specifically, there was no significant difference in the median time to the first recurrence between the locoregional and distant recurrence groups for all patients (*P* = 0.315), the ypN- group (*P* = 0.534), and the ypN+ patients (*P* = 0.904) (**Figure 5C**). Furthermore, the survival probability was similar between the locoregional and distant recurrence groups in the ypN- group (*P* = 0.942) and in the ypN+ group (*P* = 0.234) after recurrence (**Figure 5D**).

As mentioned above, a significant difference existed in the median time to the first recurrence between the ypN- group and the ypN+ group. Recurrence timing and frequency according to ypN status were summarized in **Table 4**, which showed the difference mainly originated from the rate of locoregional recurrence within the first year. Recurrence rates within the first year were 65.9% (29/44) and 85.2% (46/54) in the ypN- and ypN+ groups, respectively (*P* = 0.025). This was due to a higher locoregional recurrence rate within the first year in the ypN+ group (ypN- vs. ypN+: 63.6% vs. 94.7%, *P* = 0.028) (**Table 4**).

**Table 4:** Recurrence timing and frequency for each recurrence pattern based on lymph node status.

Recurrence	Number	0-12.0 months	12.1-24.0 months	24.1-36.0 months	36.1-48.0 months	Median (months)
All	44	29 (65.9%)	10 (22.7%)	4 (9.1%)	1 (2.3%)	7.8 (1.2,36.8)
Locoregional	11	7 (63.6%)	2 (18.2%)	2 (18.2%)	0 (0.0%)	7.8 (3.2,32.0)
Distant	33	22 (66.7%)	8 (24.2%)	2 (6.1%)	1 (3.0%)	7.8 (1.2,36.8)
All	54	46 (85.2%)	4 (7.4%)	1 (1.9%)	3 (5.6%)	5.8 (0.9,46.5)
Locoregional	19	18 (94.7%)	0 (0.0%)	1 (5.3%)	0 (0.0%)	5.8 (0.9,32.4)
Distant	35	28 (80.0%)	4 (11.4%)	0 (0.0%)	3 (8.6%)	5.6 (1.1,46.5)

## 4 Discussion

This study unveiled several interesting findings. Firstly, ypN status was associated with gender, alcohol history, clinical stage, TRG, and lymphovascular and perineural invasion. The ypN- group had lower rates of overall recurrence, locoregional recurrence, and distant metastasis when compared to the ypN+ group. In the recurrent subgroup, the extended OS in the ypN- group compared to the ypN+ group originated from the time intervals between surgery to recurrence and recurrence to death. However, we found the recurrence pattern was similar between the ypN- and ypN+ groups, regardless of the binary classification of recurrence pattern or specific site involvement, except for brain metastasis. In addition, locoregional and distant recurrence had no impact on recurrence timing and prognosis in the recurrent population and within the ypN- or ypN+ subgroups.

The NCCN guideline indicates that at least 15 lymph nodes should be removed and assessed to achieve adequate nodal staging in patients undergoing esophagectomy without induction chemoradiation (12). As the value of lymphadenectomy after nCRT for esophageal cancer is debated, the optimum number of nodes after preoperative chemoradiation is unknown. Patients from the CROSS trial showed that the number of resected nodes was not associated with survival (13). However, a large population-based cohort study of 2698 patients (including the patients who participated in the CROSS trial) demonstrated an association between LND and OS (14). Besides, reanalysis of NEOCRTEC5010 trial also showed higher number of LND associated with better survival and less recurrence in patients receiving surgery after nCRT, without increasing the risk of post-operative complications (15). Therefore, systemic lymphadenectomy is adopted in our hospital to achieve similar LND. Although a small proportion of patients had fewer than 15 lymph node dissections (<10%), the reduced number of LND contributed to the effect of induction, rather than to a less optimal nodal dissection during surgery after nCRT (15).

The status of lymph nodes serves as an indicator of the extent of disease response to nCRT and multiple reports have demonstrated its association with recurrence and prognosis in ESCC patients (4, 10). However, limited literature researched on the correlation between ypN status and clinicopathological factors. Hsu PK et al. divided pathologic lymph node regression (LNR) into three levels according to tumor involvement in the lymph nodes retrieved during esophagectomy. They found complete LNR was significantly associated with lower risks of lymphovascular and perineural invasion, as well as a higher rate of female and TRG0. Moreover, they also found that ypN and LNR were linearly dependent in this study (16). Hence, it was understandable from our findings that the ypN status exhibited associations with gender, alcohol history, clinical stage, TRG, and lymphovascular and perineural invasion.

The CROSS and NEOCRTEC5010 clinical trials both showed that nCRT can reduce locoregional and distant recurrence (1, 17). The NEOCRTEC5010 study, which involved nCRT followed by surgery in a total of 184 ESCC patients, reported a recurrence rate of 33.7% (62/184), with 29.0% (18/62) experiencing locoregional recurrence and 71.0% (44/62) patients experiencing distant metastasis (17). Our data aligned with the NEOCRTEC5010 study findings, indicating a recurrence rate of 33.3% (103/309), with 29.1% (30/103) locoregional recurrence and 66.0% (68/103) distant metastasis. The remaining 4.9% (5/103) of cases had an unknown site of recurrence. In addition, Leng X et al. reanalyzed the NEOCRTEC5010 study and found that patients with ypN+ in the nCRT group had decreased OS and RFS compared to ypN- patients, which means the lower recurrence rate in the ypN- group translated into a survival advantage (18). This also applied to the population experiencing recurrence. After categorizing the recurrent population based on lymph node status, we found that both the rates of locoregional and metastasis recurrence were higher in ypN+ patients with recurrence compared to ypN- patients with recurrence. Similarly, the OS in the ypN- group was significantly prolonged compared to the ypN+ group.

Apart from the correlation between lymph node metastasis and recurrence rate as well as prognosis, the NEOCRTEC5010 trial also identified pN1 stage as an independent risk factor for early postoperative recurrence for overall patients who received surgery with or without nCRT (19). Our research showed that ypN status was associated with the time interval between surgery to the first recurrence in the recurrent population (ypN- vs. ypN+: 7.8 months vs. 5.8 months, *P* = 0.0056). Sun Z et al. also identified ypN metastasis was an independent predictor of RFS, which was consistent with our finding (20). The median time to the first recurrence for patients who experienced recurrent disease was 6.8 months, whereas this value was 10.8 months in the NEOCRTEC5010 trial (17). The NEOCRTEC5010 trial used the AJCC 6th edition staging, which only categorized cN as cN0 and cN1 without providing information on the number of lymph node metastasis (21). In contrast, we used the AJCC 8th edition staging, which allowed for a more detailed classification of the N category. In our sample, approximately 50% of patients were classified as N2 and N3, suggesting a high proportion of advanced stage patients in our study population (22). In another real-world analysis, the median time to the first recurrence (7.7 months) was similar to our finding (5).

The discussion on recurrence pattern of ESCC patients receiving nCRT followed by surgery mainly focused on the following aspects: recurrence pattern in all patients undergoing nCRT plus surgery (23); different recurrence patterns after nCRT compared to surgery alone (17, 24); variations in recurrence pattern between patients who achieved pCR and those who did not after nCRT plus surgery (25); and recurrence pattern in patients who achieved pCR after nCRT plus surgery (7, 8, 26). We conducted a separate analysis of the recurrence pattern in the population that experienced recurrence and incorporated the lymph node status into the analysis. Nevertheless, we observed that the proportion of recurrence was comparable between the ypN- and ypN+ groups, regardless of the dichotomies of recurrence pattern or the involvement of specific sites with the exception of brain metastasis.

Moreover, the NEOCRTEC5010 trial found that patients with distant metastasis and those with locoregional recurrence shared comparable post-recurrence survival and 5-year OS after undergoing esophagectomy with or without preoperative nCRT (19). When the population was specifically defined as patients who experienced postoperative recurrence after receiving nCRT, Nagaki Y et al. also found no significant differences in the median time to first recurrence or OS among patients with locoregional or distant recurrence (6). This result was consistent with our own findings. Furthermore, we observed that locoregional and distant recurrence did not affect the timing of recurrence and prognosis within the ypN- or ypN+ subgroups.

In addition, lymph node metastasis was associated with the effect of adjuvant therapy. In adenocarcinoma, patients with residual nodal disease initially showed improved survival with adjuvant chemotherapy (27). The survival benefit of conventional adjuvant therapy (chemotherapy and/or radiotherapy) for ESCC patients was small, possibly only effective for ypN2-3 and ypT3-4 disease (9). Immunotherapy was proved to be a groundbreaking option for adjuvant therapy with the success of the CheckMate 577 trial, which showed adjuvant nivolumab immunotherapy doubled median disease-free survival in resected esophageal or gastroesophageal junction cancer with residual pathological disease (ypT+/ypN+) after nCRT (28). In other words, the ypN status served as a guide for the utilization of adjuvant therapy.

Our study had several limitations. Firstly, the results may be influenced by its retrospective nature, which could introduce selection bias. Restrained by the limited number of recurrent patients, we did not analyze whether the total number of positive nodes would have affected recurrence pattern in terms of recurrence time and prognosis. In addition, the treatments provided after recurrence varied among the patients, including chemotherapy, radiotherapy, and immunotherapy, which we did not address in our analysis.

## 5 Conclusion

Lymph node metastasis was associated with unfavorable clinicopathological factors and high risks of recurrence. Although the recurrence pattern was similar between the ypN- and ypN+ groups in the recurrent subgroup, ypN+ patients with recurrence experienced earlier relapse and had a poorer prognosis than ypN- patients with recurrence. Lymph node status might be a useful predictor of recurrence timing and prognosis, which can be valuable in formulating a strategy for perioperative treatment to improve ESCC patient survival.

## Data availability statement

The raw data supporting the conclusions of this article will be made available by the authors, without undue reservation.

## Ethics statement

The studies involving humans were approved by the Institutional Review Board of West China Hospital (2023-1343). Informed Consent Statement: The studies were conducted in accordance with the local legislation and institutional requirements. The ethics committee/institutional review board waived the requirement of written informed consent for participation from the participants or the participants’ legal guardians/next of kin because this study was only an observational study and no intervention measures were taken against the researchers.

## Author contributions

GL: Conceptualization, Data curation, Formal analysis, Investigation, Methodology, Validation, Visualization, Writing – original draft, Writing – review & editing. BH: Data curation, Methodology, Supervision, Writing – original draft, Writing – review & editing. TC: Investigation, Validation, Visualization, Writing – original draft. XZ: Formal analysis, Supervision, Writing – original draft. YT: Data curation, Investigation, Writing – original draft. QC: Formal analysis, Validation, Writing – review & editing. HS: Conceptualization, Funding acquisition, Writing – review & editing. 
